# *H. pylori* Eradication Therapy

**DOI:** 10.1155/2013/935635

**Published:** 2013-02-21

**Authors:** Ping-I Hsu, Nan-Jing Peng

**Affiliations:** ^1^Division of Gastroenterology, Department of Internal Medicine, Kaohsiung Veterans General Hospital and National Yang-Ming University, Kaohsiung, Taiwan; ^2^Department of Nuclear Medicine, Kaohsiung Veterans General Hospital and National Yang-Ming University, 386 Ta-Chung 1st Road, Kaohsiung 813, Taiwan

As a general rule for the treatment of infectious diseases, clinicians should prescribe anti-*H. pylori* regimens that have a per-protocol eradication rate ≥90%. However, the eradication rate of the standard triple therapy recommended by most guidelines has generally declined to unacceptable levels (i.e., 80% or less) recently. The reasons for this fall in efficacy with time are uncertain but may relate to the increasing incidence of clarithromycin-resistant strains of *H. pylori, *poor compliance, and rapid metabolism of proton pump inhibitor (PPI) [1, 2]. Recently, several treatment regimens have emerged to cure *H. pylori *infection. The novel first-line anti-*H. pylori* therapies include sequential therapy [3], concomitant quadruple therapy [4], hybrid (dual-concomitant) therapy [5], and bismuth-containing quadruple therapy. 

After the failure of standard triple therapy, a bismuth-containing quadruple therapy regimen comprising a PPI, bismuth, metronidazole, and tetracycline as a second-line therapy is recommended. Recently, a triple therapy with the combination of a PPI, levofloxacin, and amoxicillin has been proposed as an alternative to the standard rescue therapy and can achieve a higher eradication rate than a bismuth-containing quadruple therapy in some regions. Most guidelines suggest that patients requiring a third-line therapy should be referred to medical center and treated according to the antibiotic susceptibility test. However, it has been reported that the sensitivity of culture is less than 60%. Additionally, in vitro antimicrobial sensitivity does not necessarily lead to eradication in vivo and vice versa.

The main focus of the special issue is on the recent advances in the treatment of *H. pylori *infection. This special issue reviews the novel first-line eradication regimens with a per-protocol eradication rate exceeding 90%. In addition, the emerging rescue therapies for the second-line and third-line therapies are also discussed. 

In the paper entitled “*Pathogenesis of Helicobacter pylori-related gastroduodenal diseases from molecular epidemiological studies*,” Y. Yamaoka presents African and Asian enigmas regarding high prevalence of *H. pylori *infection and low incidence of gastric cancer. This discrepancy could be explained in part by different types of *H. pylori* virulence factors, especially CagA, VacA, OipA, and DupA.

In the paper entitled “*Recent insights into antibiotic resistance in Helicobacter pylori eradication*,” W. Wu et al. present the antibiotic resistance rates in different continental areas and the impact of antibiotic resistances on the eradication of *H. pylori*. 

In the paper entitled “*Variability in prevalence of Helicobacter pylori strains resistant to clarithromycin and levofloxacin in Southern Poland*,” E. Karczewska et al. compared the primary and secondary resistance of *Helicobacter pylori *strains isolated between 2006–2008 and 2009–2011 to clarithromycin and levofloxacin in Southern Poland. The data indicated the increasing amount of resistant *H. pylori *strains isolated from patients in Southern Poland to levofloxacin, with a simultaneous decreasing number of resistant strains to clarithromycin.

In the paper entitled “*The optimal first-line therapy of Helicobacter pylori infection in year 2012*,” C.-H. Kuo et al. review the literature about first-line therapies for *H. pylori *infection in the recent years. The efficacies of emerging first-line therapies including sequential therapy, concomitant therapy and hybrid therapy are well assessed. 

In the paper entitled “*Helicobacter pylori eradication therapies in the era of increasing antibiotic resistance: a paradigm shift to improved efficacy,*” S. D. Georgopoulos et al. present critical issues regarding the currently available means for the management of *H. pylori *infection. The existing evidences of their clinical validation and widespread applicability are also discussed. 

In the paper entitled “*7-day nonbismuth-containing concomitant therapy achieves a high eradication rate for Helicobacter pylori in Taiwan,*” S.-S. Kao et al. report that 7-day concomitant therapy achieves a very high eradication rate for *H. pylori *infection in Taiwan. The novel therapy is well tolerated. Drug compliance is an important clinical factor influencing its treatment efficacy.

In the paper entitled “*Comparison between single-dose esomeprazole- and pantoprazole-based triple therapy on the effectiveness for Helicobacter pylori eradication in Taiwanese population,*” H.-Y. Shih et al. show a higher eradication rate in esomeprazole containing triple therapy than pantoprazole containing triple therapy. The incidence of adverse effects and the compliance between two therapies are comparable.

In the paper entitled “*Rescue therapy for Helicobacter pylori infection 2012,*” J. P. Gisbert reviews current rescue therapies for *H. pylori *infection. He suggests that the choice of a “rescue” treatment depends on which treatment is used initially. If a first-line clarithromycin-based regimen was used, a second-line metronidazole-based treatment (quadruple therapy) may be used afterwards, and then a levofloxacin-based combination would be a third-line rescue option. Alternatively, a quadruple regimen may be reserved as a third rescue option if levofloxacin-based combination is used as a second-line therapy.

In the paper entitled “*Impact of Lactobacillus reuteri supplementation on anti-Helicobacter pylori levofloxacin-based second-line therapy*,” V. Ojetti et al. assessed the efficacy of *L. reuteri *supplementation in *H. pylori *eradication and in preventing gastrointestinal side effects during a second-line levofloxacin triple therapy. The data indicate that *L. reuteri *supplementation increases the eradication rate while reducing the incidence of the most common side effects associated with antibiotic therapy in a second-line treatment.

In the paper entitled “*Culture method and PCR for the detection of Helicobacter pylori in drinking water in Basrah Governorate Iraq,*” A. A. Al-Sulami et al. examined the isolated *H. pylori *from drinking water in Basrah, Iraq, on modified Columbia urea agar and HP media using the MDCS method and then confirmed that by conventional biochemical tests and 16S. rRNA PCR. The data indicate that isolating *H. pylori *from drinking water, tap and reverse osmosis water samples, by the culture method and consequent identification by biochemical tests and PCR represent a clear signal for the presence of this dangerous pathogen in the consumable water.

In the paper entitled “*A real world report on intravenous high-dose and non-high-dose proton-pump inhibitors therapy in patients with endoscopically treated high-risk peptic ulcer bleeding*,” L.-S. Lu et al. conducted a retrospective case-controlled study to investigate the real world experiences in prescribing high-dose and non-high-dose proton-pump inhibitor therapy for preventing rebleeding after endoscopic treatment of high-risk peptic ulcer bleeding, a life-threatening complication of *H. pylori*-related disease. This study suggests that the effect of intravenous high-dose pantoprazole may not be superior to non-high-dose regimen in reducing rebleeding in high-risk peptic ulcer bleeding. However, selection bias may exist in a high-dose group caused by clinicians' decision on PPI dosage in patients with more severe diseases or with less manageable bleeding ulcers.

*Ping-I Hsu* *Ping-I Hsu* *Nan-Jing Peng* *Nan-Jing Peng*

## References

[1] Chuah SK, Tsay FW, Hsu PI, Wu DC (2011). A new look at anti-*Helicobacter pylori* therapy. *World Journal of Gastroenterology*.

[2] Graham DY, Akiko S (2008). New concepts of resistance in the treatment of *Helicobacter pylori* infections. *Nature Clinical Practice Gastroenterology & Hepatology*.

[3] Zullo A, De Francesco V, Hassan C, Morini S, Vaira D (2007). The sequential therapy regimen for *Helicobacter pylori* eradication: a pooled-data analysis. *Gut*.

[4] Wu DC, Hsu PI, Wu JY (2010). Sequential and concomitant therapy with four drugs is equally effective for eradication of *H. pylori* infection. *Clinical Gastroenterology and Hepatology*.

[5] Hsu PI, Wu DC, Wu JY, Graham DY (2011). Modified sequential *Helicobacter pylori* therapy: proton pump inhibitor and amoxicillin for 14 days with clarithromycin and metronidazole added as a quadruple (Hybrid) therapy for the final 7 days. *Helicobacter*.

